# Depression and Health-Related Quality of Life among Patients with Type 2 Diabetes Mellitus: A Cross-Sectional Study in Nepal

**DOI:** 10.1371/journal.pone.0141385

**Published:** 2015-11-23

**Authors:** Shiva Raj Mishra, Abhishek Sharma, Parash Mani Bhandari, Shristi Bhochhibhoya, Kiran Thapa

**Affiliations:** 1 Nepal Development Society, Bharatpur 10, Chitwan, Nepal; 2 Department of Global Health, Boston University School of Public Health, Boston, Massachusetts, United States of America; 3 Center for Global Health and Development, Boston University School of Public Health, Boston, Massachusetts, United States of America; 4 Institute of Medicine, Maharajgunj Medical Campus, Tribhuvan University, Kathmandu, Nepal; National Institute of Health, ITALY

## Abstract

**Background:**

Diabetes is accompanied by a marked reduction in patient’s quality of life (QOL) and leads to higher disability-adjusted life years than most diseases. Depression further deteriorates QOL and is associated with poor treatment outcomes and lowered glycemic control in diabetes. We analysed the QOL and depression among the people living with diabetes in Nepal.

**Methods:**

We conducted a cross-sectional survey among a random sample of 157 diabetic patients visiting diabetes clinic at a major teaching hospital in Kathmandu, Nepal. We administered the Nepali version of WHO-BREF for face to face interviews to obtain data on QOL scores. The Nepali version of Patient Health Questionnaire-9was also used to record responses on depression items.

**Results:**

More than half of the respondents (54.1%) experienced depression with mean PHQ-9 score of 6.15 ± 5.01 on a scale of 0–27. On a scale of 0 to 100, highest QOL mean score was reported in social relationship domain (57.32 ± 11.83), followed by environment domain (54.71 ± 7.74), psychological health (53.25 ± 10.32) and physical health (50.74 ± 11.83). After adjusting for other covariates, urban residence decreased the physical health score by 4.74 (β = -4.74, 95% CI: -8.664,-0.821), social relationship domain score by 3.420 (β = -3.420, 95% CI: -6.433,-0.406) and the overall QOL by 2.773 (β = -2.773, 95% CI: -5.295,-0.252). Having diagnosed with diabetes since more than 10 years increased physical health by 5.184 score points (β = 5.184; 95% CI: 0.753, 9.615).Similarly, having severe depression decreased social relation domain score by 6.053 (β = -6.053, 95% CI:-11.169,-.936).

**Conclusion:**

Having urban residence significantly decreased the physical health and social relation domain scores as well as the overall QOL scores. Similarly, having diagnosed since more than 10 years increased physical health domain score. Severe depression decreased social relationship domain score. Since depression affects QOL, we suggest early diagnosis and prompt treatment of depression in T2DM people as part of their routine primary care in Nepal.

## Introduction

The increasing burden of diabetes among adults (aged 20–70 years) is a major public health concern globally [1].The number of people living with diabetes is estimated to rise from 135 million in 1995 to 300 million by 2025. Developing countries are expected to observe a 170% increase in prevalence of diabetes compared to 42% increase in developed countries. In 2025, over 75% of people with diabetes globally will be residing in developing countries [2].In particular the diabetes prevalence among Asian populations is increasing rapidly, driven largely by economic development, nutrition transition, and sedentary lifestyles. In 2007, around 110 million individuals were living with diabetes in Asia, with young and middle aged population being affected disproportionately [3].

Nepal, largely an agrarian country, has witnessed a rapid surge of diabetes cases in recent years. According to the World Health Organization (WHO) estimates, the cases of diabetes in Nepal are expected to increase from the 436,000 (2% prevalence) in 2000 to 1,328,000 (10% prevalence) in 2030 [4]. Baral et al reported the prevalence of diabetes at 5.3% [5].There exists a rural-urban divide in diabetes prevalence: while 2.5% of the rural populations are living with diabetes, the prevalence is as high as 14.6% among the urban population [6]. Kathmandu, the capital and largest metropolitan city in Nepal has diabetes prevalence of 25.9% [7].

Type 2 diabetes mellitus (T2DM) accounts for the majority of all diabetes cases. T2DM has a number of chronic effects, including disability, cardiovascular disease, kidney disease, and blindness [1].T2DM is also accompanied by marked reduction in the quality of life (QOL) [8]. Comorbid depression further reduces QOL in people with T2DM [9], and is associated with poor treatment outcomes and lowered glycemic control [10, 11]. Prevalence of depression is twofold in people with T2DM compared to those without [12]. In Nepal, the prevalence of depression among people living with diabetes (40.3%) is more than twice the global prevalence of 17.6% [10–11]. This suggests that people with T2DM in Nepal may have relatively lower QOL. While several studies have addressed the issue of QOL among diabetes patients [8, 13–16] across different cultural settings globally, a little is known about the QOL among the T2DM patients in Nepal. Understanding which QOL domains are associated with depression among patients with diabetes in Nepal will be useful for better diabetes management in clinical settings. To that end, we analyzed the QOL scores among people with T2DM in Nepal. This study was conducted prior to the unfortunate Nepal earthquakes in early 2015 which had devastating effects on healthcare service delivery system and population’s lifestyle [17].

## Methods

### Study design and tools

We conducted a cross sectional study to assess the QOL among patients living with T2DM in Nepal. We employed the WHOQOL-BREF, a shorter version of WHOQOL 100 survey tool, which was developed by the WHO to capture the broad aspects of health related QOL [18].Through field trials in many countries, WHOQOL-BREF has been validated as a standard tool to measure QOL cross culturally. In particular, we employed the Nepali version of WHO-BREF as used by Giri et al [19].This survey tool is a structured questionnaire consisting of 26 items in four domains, namely i) physical health, ii) psychological health, iii) social relationship, and iv) environment. Tables 1 and 2 summarizes the features of WHOQOL-BREF. Higher scores denote higher QOL. We also included nine additional Nepal specific socio-demographic variables in our questionnaire, namely history of diagnosis (time since diagnosis of diabetes as of the day of survey), current drinking (alcohol) status, smoking status, consumption of any tobacco product, depression, diabetes complications such as retinopathy, neuropathy, cardiac complications and nephropathy. The questionnaire and scoring methods on WHO-BREF are described elsewhere [18]. We used Nepali version of Patient Health Questionnaire-9 (PHQ-9), a concise and self-administered tool for screening and diagnosis of depression. The tool has been well-validated with sufficient sensitivity and specificity for the diagnosis of major depression [20, 21]. The tool has nine items for recording frequency of symptoms of depression during the past 2 weeks. Table 2 summarizes the components of PHQ-9. The responses are recorded as ‘not at all’, ‘several days’, ‘more than half the days’ or ‘nearly every day’. Patients identified with major depression disorder were advised for further medical assistance. Nepali version of both the WHO-BREF and PHQ9 were pre-tested separately among 20 diabetes patients to test its accuracy before data collection. Based on pre-testing, we made minor changes in wording of the questions.

**Table 1:** Summary of WHOQOL-BREF survey components [19].

Domain	Features
Physical	Activities of daily living
	Work capacity
	Energy and fatigue
	Pain and discomfort
	Dependence on medicinal substances/aids
	Sleep and rest
Psychological	Bodily image and appearance
	Negative feeling
	Positive feeling
	Self-esteem
	Thinking, learning. Memory and concentration
Social relations	Personal relationships
	Social support
	Sexual activity
Environment	Financial resources
	Freedom, physical safety and security
	Health and social care: accessibility and quality
	Home environment
	Opportunities for acquiring new information and skills
	Participation opportunities for recreation and leisure
	Physical environment (pollutant, noise/traffic/climate)
	Transport

**Table 2:** Summary of PHQ-9 components.

Interest or pleasure in activity
Feeling down, depressed, or hopeless
Trouble falling or staying asleep or sleeping too much
Feeling tired or having little energy
Poor appetite or overheating
Feeling bad about yourself or low self-esteem
Trouble concentrating on reading, recreation or work
Talking to yourself or restlessness
Suicidal ideation or self-harm

Diabetes is often accompanied by overweight and obesity, so measuring BMI and checking for overweight or obesity status can further help explore QOL in people with diabetes. Therefore we also conducted anthropometric measurement of the participants' height and weight. Height of the respondents was measured in standing position using portable stadiometer to the nearest centimeters. Weight was measured by using bathroom scale, calibrated at zero initially, which could detect a mass to nearest 0.1 kg. We measured the waist circumference at midway between the lowest rib and the iliac crest to the nearest 1 cm using a tailors’ (inch) tape. For each anthropometric measure, we took two readings for a given patient and the average values were considered as final.

### Study setting and sample

Tribhuvan University Teaching Hospital (TUTH) is the largest tertiary hospital in Nepal. It is located in the metropolitan city of Kathmandu. Attended by thousands of patients daily, this hospital is a major source of affordable specialty care for people living in Kathmandu valley as well as those in distant rural areas of Nepal. Many people get tested for diabetes voluntarily, or are referred by other departments of TUTH. The hospital has one diabetes care clinic with several staff members that provides services including glycemic monitoring and counselling.

Based on the hospital informal statistics, an average of 30 people visited the diabetes clinic daily during the month of April 2014. Between July and September 2014, trained interviewers recruited every fifth patients visiting the clinic (i.e. approximately 6 people a day) and conducted face-to-face interviews at the clinic. We recruited 157 participants by the end of the study. Interviewers were trained in interviewing techniques and had been involved in similar research projects previously.

### Definition of variables

#### Outcome variables

The participant scores on the four domains of WHO-BREF (see Table 1) constituted the outcome variables. The scores on the 26-item questions were initially measured on a scale of 4–20 initially, and then these were converted to a scale of 100 in order to make the results comparable to any studies which employ the WHOQOL-100 questionnaire.

#### Independent variables

We collected the information about age, gender, ethnicity, education, occupation, marital status, monthly income (<10,000 NPR, 10,000–24,000 NPR, >24,000 NPR), residence (rural, urban), family history of diabetes (Yes, No), years since diagnosis (≤10 years, >10 years), use of any form of tobacco (Yes, No), current drinking (Yes, No), waist circumference (High if >90 cm, Low if ≤90 cm) and BMI (kg/m^2^) (<25, 25–30,>30). The respondents who resided at a Village Development Committee were classified as rural inhabitants whereas those residing in a municipality were classified as urban inhabitants. Having any diabetes complication was reported "yes” if respondents reported at least one of the complications i.e. cardiac complications, retinopathy, neuropathy and nephropathy. Diabetic complications were assessed by the interviewers on the basis of symptoms reported by the patients. PHQ-9 score was measured as a continuous variable initially; however it was also categorized into ‘no depression’ (0–4), ‘mild’ (5–9), ‘moderate’ (10–14) and ‘severe depression’ (≥ 15), which was further categorized into ‘no severe depression (<10)’and ‘severe depression (≥ 10)’ during regression analysis.

Another important factor affecting diabetes patients’ QOL is their compliance to the ongoing therapy, which we could estimate by measuring patients’ glycated haemoglobin (HbA1c) levels. However given the resource constraints to conduct this survey, we did not measure HbA1c levels.

### Data analysis

We entered the collected data in EpiData 3.1 and then transferred the data to SPSS IBM 20 software for analysis. Descriptive statistics were presented as frequency, percentage, mean and median. Pearson’s correlation, chi-square test and analysis of variance (ANOVA) tests were used for categorical, discrete and continuous variables. Variables with p-value<0.10 (alpha significance level) were further analyzed using multivariate analysis using a stepwise backward elimination procedure.

To assess the internal consistency (i.e. the extent to which different domain items lead to consistent results) of the survey tool, we performed Cronbach alpha test. We also tested the correlation between individual domain scores.

### Ethical approval

We obtained ethical approval to conduct this study from the institutional review committee of Manmohan Memorial Institute of Health Sciences, Lalitpur, Nepal. The study objectives were clearly explained to the study participants prior to the data collection and their written consents were obtained. Respondents were informed about their right to not answer any questions or to withdraw at any time during the interview.

## Results

### Demographic characteristics of study participants

Table 3 presents the participant's demographic characteristics. 60.5% of the participants were female, 41.4% were 45–59 years of age, 49.7% belonged to Brahmin/Chhetri ethnic group and 59.9% had completed 10 or less years of schooling. Nearly two third (65.6%) of the participants resided in urban areas, 51.6% were unemployed and 45.6% had monthly income of NPR 10,000–24,000 (100 NPR = 1 USD). More than two third of the respondents (77.1%) were living with diagnosed diabetes for 10 years or less. Few respondents were smokers (5.7%) or consumed alcohol (8.3%) or any form of tobacco products (12.7%). Nearly 40.1% of respondents had positive family history of diabetes. Over half (54.1%) of the respondents experienced depression: of which 30.6% of the respondents had mild depression and 8.3% were severely depressed. Regarding the co-morbidities, retinopathy (26.8%), neuropathy (32.5%), cardiac complications (14.6%) and nephropathy (1.3%) were present among the respondents.

**Table 3:** Demographic characteristics of the study participants.

Characteristics	Frequency (N = 157)	%
**Gender**		
Male	62	39.5
Female	95	60.5
**Age** (Mean ± SD: 53.14 ± 11.53 years)		
30–44 years	43	27.4
45–59 years	65	41.4
> = 60 years	49	31.2
**Ethnicity**		
Brahmin/Chhetri	78	49.7
Aadibasi/Janajati	64	40.8
Others	15	9.6
**Education**		
Illiterate	34	21.7
Upto 10 years of schooling	94	59.9
Higher	29	18.5
**Residence**		
Rural	54	34.4
Urban	103	65.6
**Marital status**		
Unmarried	2	1.3
Ever married	155	98.7
**Employment**		
Employed	76	48.4
Unemployed	81	51.6
**Family income** (Mean ± SD: 2000 ± 21,555.05 NPR)		
<10000 NPR	23	14.6
10000–24000 NPR	72	45.6
>24000 NPR	62	39.5
**Years since diabetes diagnosis** (Mean ± SD: 6.89 ± 6.66 years)		
≤10 years	121	77.1
>10 years	36	22.9
**Current drinking (alcohol) status**		
No	144	91.7
Yes	13	8.3
**Current smoking status**		
No	148	94.3
Yes	9	5.7
**Consumption of any form of tobacco**		
No	137	87.3
Yes	20	12.7
**Family history of diabetes**		
No	94	59.9
Yes	63	40.1
**Severity of depression** (Mean±SD: 6.15±5.01)		
None	72	45.9
Mild	48	30.6
Moderate	24	15.3
Severe	13	8.3
BMI (kg/m^2^)		
<25	83	52.9
25–30	65	41.4
>30	9	5.7
Retinopathy		
No	115	73.2
Yes	42	26.8
**Neuropathy**		
No	106	67.5
Yes	51	32.5
**Cardiac**		
No	134	85.4
Yes	23	14.6
**Nephropathy**		
No	155	98.7
Yes	2	1.3

### Measurement properties of WHO-BREF

We found good internal consistency of the WHO-BREF questionnaire among the diabetes patient population. Cronbach’s alpha was 0.872 for the WHO-BREF questionnaire and was 0.756 between the individual domains scores (internal consistence), which is fairly higher than the usual standard of 0.7.Cronbach’s Alpha is the measurement of scale reliability. Higher value of it indicates higher internal consistency and vice versa. It is the function of number of test items and average inter item correlation. More on this with practical computing examples is explained elsewhere [22].The Pearson’s correlation coefficients between domain scores ranged from 0.327 to 0.827, thereby indicating a fairly good correlation overall. See Appendix for the correlation coefficients in four domains of WHO-BREF.

### Factors associated with health-related QOL scores

Table 4 summarizes the domain-specific QOL scores among the study population. We found the highest mean (SD) score in social relationship domain (57.32 ± 8.94), followed by environment domain (54.71 ± 7.74), psychological health (53.25 ± 10.32) and physical health (50.74 ± 11.83). The Kolmogorov-Smimov normality test scores showed normal distribution in case of all domains and therefore we performed parametric hypothesis testing with the alpha significance level of 0.10. The findings from analysis of mean difference by covariates are shown in Table 5. BMI and waist circumference did not have any association with individual domain scores and overall QOL score. We found significant difference by residence status in physical health domain (p-value = 0.055), years since diabetes diagnosis in physical health domain (p-value = 0.068) and depression in social relationship domain (p-value = 0.031) and neuropathy in physical health (p-value = 0.019), and psychological health domain (p-value = 0.078).

**Table 4:** Quality of life (QOL) domain scores among the study participants.

Domains	Min	Max	Mean (SD)
Physical health	18.29	80.00	50.74(11.83)
Psychological health	26.67	77.33	53.25(10.32)
Social relationship	32.00	80.00	57.32(8.94)
Environment	34.00	76.00	54.71(7.74)
Overall QOL	31.74	74.05	54.01(7.47)

**Table 5:** Bivariate association between independent variables and quality of life domains.

Characteristics	Physical Health (Mean(SD))	Psychological (Mean(SD))	Social Relationship (Mean(SD))	Environment (Mean(SD))	Overall QOL (Mean(SD))
**Age**					
Coefficient	0.057	0.073	-0.039	-0.044	0.025
p-value	0.48	0.36	0.63	0.58	0.76
**Gender**					
Men	49.92(12.63)	52.13(10.72)	56.77(9.23)	54.68(7.57)	53.37(7.54)
Women	50.45(11.34)	53.47(10.31)	57.38(9.03)	54.67(7.90)	53.99(7.56)
p-value	0.78	0.43	0.69	0.99	0.62
**Ethnicity**					
Brahmin/Chhetri	50.52(13.04)	51.97(10.76)	57.50(9.44)	54.26(7.84)	53.56(8.10)
Janajati/Aadibasi	50.36(10.66)	54(10.63)	56.58(8.43)	54.59(8.12)	54.01(7.39)
Others	48.30(10.43)	51.20(7.28)	57.60(10.51)	57.68(7.74)	53.58(4.99)
p-value	0.80	0.28	0.82	0.40	0.93
**Education**					
Illiterate	52.44(10.43)	56.47(8.34)	57.88(7.33)	55.00(7.45)	55.45(6.32)
Upto 10 years of schooling	49.24(11.85)	51.86(10.99)	57.13(9.14)	54.66(7.54)	53.22(7.66)
Higher	50.91(13.23)	52.32(10.31)	56.28(10.91)	54.34(8.92)	53.46(8.34)
p-value	0.38	0.082	0.77	0.95	0.33
**Occupation**					
Employed	50.55(12.30)	53.40(10.44)	57.12(9.33)	54.87(7.25)	53.99(7.54)
Unemployed	49.95(11.44)	52.51(10.52)	57.15(8.93)	54.49(8.21)	53.53(7.57)
p-value	0.75	0.59	0.98	0.76	0.70
**Marital status**					
Unmarried	56.00(1.62)	60.00(1.89)	56.00(11.31)	57.00(4.24)	57.25(0.89)
Ever married	50.17(11.89)	52.85(10.49)	57.15(9.11)	54.65(7.78)	53.70(7.57)
p-value	0.49	0.34	0.86	0.67	0.51
**Residence**					
Rural	52.74(10.76)	53.53(10.29)	59.26(8.70)	55.67(6.94)	55.29(6.95)
Urban	48.93(12.20)	52.63(10.58)	56.03(9.14)	54.15(8.12)	52.94(7.73)
p-value	0.055*	0.611	0.034	0.25	0.062
**Monthly income**					
10000	49.39(9.64)	54.14(8.37)	58.20(5.78)	56.00(5.68)	54.43(4.18)
10000–24000	50.16(12.90)	52.66(10.91)	56.88(10.32)	54.22(8.38)	53.48(8.67)
>24000	50.65(11.40)	52.81(10.73)	57.03(8.67)	54.70(7.68)	53.80(7.15)
p-value	0.91	0.84	0.83	0.63	0.87
**Socioeconomic status**					
Upper	45.71(13.57)	48.67(8.25)	61.33(14.11)	57.50(6.19)	53.30(4.69)
Upper middle	53.51(13.39)	54.89(11.32)	56.45(9.40)	55.65(7.93)	55.13(8.63)
Middle/lower Middle	49.78(11.12)	51.68(10.46)	56.63(9.32)	53.85(8.24)	52.98(7.68)
Lower/upper lower	49.49(11.58)	53.37(10.20)	57.58(8.60)	54.75(7.39)	53.80(7.10)
p-value	0.37	0.46	0.72	0.66	0.67
**Years since diabetes diagnosis**					
10 years	49.30(11.53)	52.63(10.44)	57.08(9.28)	54.48(7.15)	53.37(7.24)
>10 years	53.39(12.44)	54.00(10.62)	57.33(8.59)	55.33(9.57)	55.02(8.45)
p-value	0.068*	0.49	0.88	0.56	0.25
**Current drinking (alcohol) status**					
No	50.41(11.86)	53.09(10.50)	57.15(9.26)	54.78(7.82)	53.86(7.62)
Yes	48.35(11.83)	51.28(10.22)	57.023(7.35)	53.54(6.98)	52.55(6.65)
p-value	0.55	0.55	0.96	0.58	0.55
**Consumption of any form of tobacco**					
No	50.45(11.84)	53.33(10.18)	57.58(8.97)	54.85(7.76)	54.05(7.35)
Yes	48.80(11.96)	50.26(12.15)	54.13(9.67)	53.50(7.756)	51.67(8.61)
p-value	0.56	0.22	0.11	0.47	0.19
**Family history of diabetes**					
No	50.21(11.65)	52.13(10.77)	56.90(8.65)	54.89(7.60)	53.79(7.68)
Yes	50.28(12.19)	52.66(10.05)	57.48(9.79)	54.35(7.99)	53.69(7.37)
p-value	0.97	0.78	0.70	0.67	0.94
**Depression**					
None	49.21(13.16)	52.25(10.21)	56.96(10.12)	72(54.53)	53.23(8.25)
Mild	50.43(11.13)	53.78(10.69)	57.00(7.95)	55.54(7.83)	54.18(7.38)
Moderate	54.38(10.06)	55.11(9.49)	60.89(6.47)	55.50(5.25)	56.47(5.10)
Severe	47.64(8.18)	49.64(12.53)	51.69(9.08)	50.77(7.51)	49.94(6.27)
p-value	0.25	0.40	0.031*	0.24	0.072*
**Number of diabetic complications**					
1 complication	51.07(11.60)	53.29(10.36)	56.59(8.85)	54.63(6.75)	53.89(7.58)
>1 complications	48.00(10.63)	51.55(8.38)	54.88(9.49)	52.17(7.71)	51.65(6.72)
p-value	0.26	0.46	0.43	0.14	0.20
**BMI (kg/m** ^**2**^ **)**					
<25	48.79(12.02)	52.56(10.44)	56.99(9.53)	54.57(7.76)	53.23(7.81)
25–29.99	52.04(11.05)	54.03(10.28)	57.92(8.05)	55.29(7.63)	54.82(6.79)
>30	50.54(14.83)	48.59(11.61)	52.74(11.75)	51.11(8.25)	59.49(3.16)
p-value	0.25	0.31	0.27	0.31	0.21
**Waist circumference**					
Low(90)	50.10(12.24)	53.00(9.96)	58.36(8.94)	55.20(7.93)	54.15(7.41)
High(>90)	50.41(11.36)	52.87(11.13)	55.57(9.11)	54.00(7.49)	53.22(7.72)
p-value	0.87	0.94	0.056	0.34	0.43
**Neuropathy**					
No	50.49(11.69)	53.11(10.40)	57.22(9.08)	54.75(7.72)	53.89(7.47)
Yes	30.86(1.62)	40.00(7.54)	50.67(11.31)	49.00(9.89)	42.63(1.94)
p-value	0.019*	0.078*	0.31	0.30	0.035

*p-value < 0.10 (significant at alpha significance level of 0.10)

Table 6 presents the result of multivariate linear regression analysis. After adjusting for other covariates, having urban residence decreased the physical health score by 4.74 scores (β = -4.74, 95% CI: -8.66, -0.82) and social relationship domain score by 3.42 scores (β = -3.42, 95% CI: -6.43, -0.41) and overall QOL by 2.77 scores (β = -2.77, 95% CI: -5.30, -0.25). Having diagnosed with diabetes since more than 10 years increased physical health by 5.18 scores (β = 5.18; 95% CI: 0.75, 9.62). Similarly, having severe depression decreased social relation domain score by 6.053 scores (β = -6.05, 95% CI: -11.17, -0.94).

**Table 6:** Multivariate linear regression analysis between independent variables and QOL scores.

Characteristics	Coefficients (95% CI)*
**Physical health**	
Residence (Urban)	-4.743 (-8.664, -0.821)
Years since diabetes diagnosis (>10 years)	5.184 (0.753, 9.615)
**Social relations**	
Residence (Urban)	-3.420 (-6.433, -0.406)
Depression (Severe depression)	-6.053 (-11.169, -0.936)
**Overall QOL**	
Residence (Urban)	-2773 (-5.295, -0.252)

*Adjusted for residence, history of diagnosis, depression and neuropathy

## Discussion

To our knowledge, this is the first study to report QOL among people with T2DM in Nepal. We found the highest average QOL score in the social relationship domain. We infer that respondents maintained fairly good relationships with friends and relatives; received fairly good support from family, community and friends, and were satisfied with their sexual life. However, the QOL scores were lowest for physical health domain which indicates lowered energy and work capacity for daily living, higher dependence on medicinal substances and medical aids; fatigue, sleep-related problems, restlessness, reduced mobility, greater pain and discomfort in daily life.

Our analysis shows that after adjusting for other variables, having urban residence was associated with decreased QOL scores in physical health and social relationship domain scores by 4.74 and 3.42, respectively, and the overall QOL reduced by 2.77 scores. While an earlier study in Iran reported no rural-urban divide in QOL [8], the decreased QOL reported among urban dwellers in our study might be because more participants are of urban origin or this pattern could be specific to Nepalese population. Having diagnosed with diabetes since more than 10 years was associated with increase in physical health domain scores by 5.18. Contrarily, earlier study reported duration since diagnosis of T2DM had no effect in physical health domain, but negative correlation with social relationship domain was observed [9].We found that severe depression decreased social relationship domain scores by 6.05. This is in accordance with earlier studies which reported a negative association between depression symptoms and at least one aspect of QOL among the T2DM patients [23].

Although we didn’t find any effect of age and education on QOL, an earlier study from Turkey has shown that education is positively correlated with physical health, psychological health, social relationship, environment, and the overall QOL [9].The same study also reported a significant negative correlation between age and social relationship domain. Similarly, while we did not find any association of gender with QOL, a study among people with diabetic foot ulcers from Iran reported that men had an overall high QOL scores in all domains compared to the women [24].Furthermore, previous literature [24][25] suggest that lower QOL scores were associated with having minor diabetes-related complications. Also the effects of age, gender, metabolic activity, and education on overall QOL were found to be weak.

Our study has few strengths and limitations. This is the first study to assess QOL and depression among diabetes patients in Nepal. We used WHO-BREF which has been widely used across cultures and regions, and is a validated tool for measuring health-related QOL worldwide. Our study was limited to a single but major healthcare center in Nepal, and therefore our findings may not necessarily be generalizable to other populations in Nepal. Also, as mentioned earlier, we did not collect any data to assess the patients’ compliance to their ongoing therapy. Previous studies found high rates of non-compliance among the diabetes patients in Nepal [26], which is likely to have adverse effects on disease progression and could lower QOL [27].However some researchers also found that depression among diabetes patients is a major risk factor for non-compliance [10,11]. While we found co-existing lower QOL and depression among the diabetes patients, we are unable to assess the effect of non-compliance. Furthermore we report the QOL among the Nepalese T2DM population prior to the devastating 2015 Nepal earthquakes which we believe may have further increased depression levels. We recommend further studies to investigate the effects of depression and other diabetes-related comorbidities on QOL among larger T2DM populations.

## Conclusion

We found that having urban residence significantly decreased physical health, social relation domain scores and overall QOL scores. Similarly, having diagnosed with diabetes since more than 10 years increased physical health domain score. We also found having severe depression significantly decreased the social relationship domain score, and might impede QOL on the whole. While literature suggests that people with diabetes have lower QOL in general, we found that depression among people with T2DM further reduced the QOL. Therefore, public health policy makers and program planners should focus on early diagnosis and prompt treatment of depression in people with diabetes as part of their routine primary care in Nepal.

## Supporting Information

S1 Table Correlation coefficients in four domains of WHO-BREF. (RTF)
