# Supplementary material for: Depression and Health-Related Quality of Life among Patients with Type 2 Diabetes Mellitus: A Cross-Sectional Study in Nepal
Source: PLoS One. 2015 Nov 23;10(11):e0141385. doi: 10.1371/journal.pone.0141385 (PMC4658137; doi:10.1371/journal.pone.0141385)
Supplement: S1 Table — (RTF) [file pone.0141385.s001.rtf]

S1 Table: Correlation coefficients in four domains of WHO-BREF 
	Physical health	Psychological health	Social relationship 	Environment 	Total quality of life 	
Physical health 	1					
Correlation coefficient 		0.646	0.396	0.343	0.827	
p-value		p<0.001	p<0.001	p<0.001	p<0.001	
Psychological health 		1				
Correlation coefficient 	0.646		0.327	0.502	0.829	
p-value	p<0.001		p<0.001	p<0.001	p<0.001	
Social relationship			1			
Correlation coefficient 	0.396	0.327		0.443	0.648	
p-value	p<0.001	p<0.001		p<0.001	p<0.001	
Environment 				1		
Correlation coefficient 	0.343	0.502	0.443		0.701	
p-value	p<0.001	p<0.001	p<0.001		p<0.001	
Total quality of life 					1	
Correlation coefficient 	0.827	0.829	0.684	0.701		
p-value	p<0.001	p<0.001	p<0.001	p<0.001		
